# Effects of jump and balance training on knee kinematics and electromyography of female basketball athletes during a single limb drop landing: pre-post intervention study

**DOI:** 10.1186/1758-2555-3-14

**Published:** 2011-07-14

**Authors:** Yasuharu Nagano, Hirofumi Ida, Masami Akai, Toru Fukubayashi

**Affiliations:** 1 Department of Health and Sports, Niigata University of Health and Welfare, Niigata, Japan; 2 Department of Human System Science, Graduate School of Decision Science and Technology, Tokyo Institute of Technology, Tokyo, Japan; 3 National Rehabilitation Center for Persons with Disabilities, Saitama, Japan; 4 Faculty of Sport Sciences, Waseda University, Saitama, Japan

## Abstract

**Background:**

Some research studies have investigated the effects of anterior cruciate ligament (ACL) injury prevention programs on knee kinematics during landing tasks; however the results were different among the studies. Even though tibial rotation is usually observed at the time of ACL injury, the effects of training programs for knee kinematics in the horizontal plane have not yet been analyzed. The purpose of this study was to determine the effects of a jump and balance training program on knee kinematics including tibial rotation as well as on electromyography of the quadriceps and hamstrings in female athletes.

**Methods:**

Eight female basketball athletes participated in the experiment. All subjects performed a single limb landing at three different times: the initial test, five weeks later, and one week after completing training. The jump and balance training program lasted for five weeks. Knee kinematics and simultaneous electromyography of the rectus femoris and Hamstrings before training were compared with those measured after completing the training program.

**Results:**

After training, regarding the position of the knee at foot contact, the knee flexion angle for the Post-training trial (mean (SE): 24.4 (2.1) deg) was significantly larger than that for the Pre-training trial (19.3 (2.5) deg) (p < 0.01). The absolute change during landing in knee flexion for the Post-training trial (40.2 (1.9) deg) was significantly larger than that for the Pre-training trial (34.3 (2.5) deg) (p < 0.001). Tibial rotation and the knee varus/valgus angle were not significantly different after training. A significant increase was also found in the activity of the hamstrings 50 ms before foot contact (p < 0.05).

**Conclusions:**

The jump and balance training program successfully increased knee flexion and hamstring activity of female athletes during landing, and has the possibility of producing partial effects to avoid the characteristic knee position observed in ACL injury, thereby preventing injury. However, the expected changes in frontal and transverse kinematics of the knee were not observed.

## Background

Anterior cruciate ligament (ACL) injury leads to knee joint instability [1] and failure to return to sports activities at the same level [2]. After ACL injury, rehabilitation can last for six months or longer before an athlete can return to sports activity [3]. Osteoarthritic changes can be found even when ACL reconstruction is performed [4]. Therefore in recent years, prevention of ACL injuries has become a key issue. Most ACL injury prevention training programs are composed of plyometrics, balance training, agility training, and instructions to avoid the characteristic stance that is slight knee flexion and forceful valgus rotation associated with ACL injury [5-10]. Although the subjects and details of the training programs are different, the results show a decrease in the incidence of ACL injury [5-9].

Some research studies have investigated the effects of training programs on knee kinematics during landing tasks; however the results were different among the studies [11-14]. An increase in knee flexion has been reported as a result of conducting plyometric training [11], plyometric or balance training [12], and videotape feedback [13]. Only Myer et al. [12] reported changes in the kinematics of the knee in the frontal plane, i.e., both plyometric and balance training decreased the knee abduction angle during a medial drop landing. In other studies [11,14], no differences in knee valgus were found after plyometric or agility training. Among all these studies, knee kinematics have only been analyzed in the sagittal and frontal plane. Even though tibial rotation is usually observed at the time of ACL injury, the effects of training programs for knee kinematics in the horizontal plane has not yet been analyzed.

Other research studies investigated the effects of training programs on electromyography during athletic tasks; however activation of mainly the hip-musculature has been reported [11,15,16]. DeMorat et al. [17] ascertained that aggressive quadriceps loading with the knee in slight flexion produces significant anterior tibial translation and internal tibial rotation and leads to ACL injury. Chappell et al. [18] reported that females tend to have greater quadriceps activation before landing than males. Pre-activation is thought to be important for the dynamic stability of the knee. Thus, it is necessary to evaluate the effects of ACL injury prevention training programs on the electromyography of the quadriceps and hamstrings of female athletes including pre-activation.

Thus, the purpose of this study was to determine the effects of a jump and balance training program on knee kinematics including tibial rotation as well as on electromyography of the quadriceps and hamstrings in female athletes. Since the training program aims at reducing ACL injury, subjects were instructed to avoid landing with their body in the characteristic position of ACL injury, which includes slight knee flexion and valgus rotation with either internal or external tibial rotation [19-21]. Furthermore, for the training program to be successful, hamstrings activation should increase to counteract against aggressive quadriceps loading. Therefore, our hypothesis was that training will increase knee flexion angle, decrease knee valgus, decrease internal tibial rotation, and increase hamstrings activation before ground contact during a single limb landing.

## Methods

### Population

Eight female basketball athletes belonging to Waseda University basketball team participated in this study. Exclusion criteria included any lower extremity reconstructive surgery in the past two years or unresolved musculoskeletal disorders that prohibited subjects from participating in sports. The average age of the subjects was 19.4 (0.7) yrs (Mean (SD)), the average height was 1.70 (0.05) m, the average weight was 64.1 (7.8) kg and the average BMI was 22.2 (1.6). Prior to participation, each subject signed a Human Subjects Informed Consent Document approved by Waseda University ethics committee.

### Experimental Task

All subjects performed a single limb drop landing from a 30 cm platform as described in a previous study [22]. The subjects were instructed to put their hands on their lower torso, stand on their right foot, and jump 30 cm away from the platform. The subjects were to land on their right foot in a neutral position. Upon landing, each subject was instructed to place their center of mass as far forward as possible in an attempt to limit horizontal motion and land without jumping up. Throughout the experiment, the subjects were barefooted to exclude the influence of differences in their shoes. Subjects were allowed several preparation trials until they could conduct the experimental task precisely. Measurement was continued until three successful trials were accomplished consecutively. We defined a failed trial when the subjects (1) could not maintain a single limb landing position, (2) landed farther than or within 30 cm of the platform, or (3) jumped up from the platform. Subjects took a sufficient rest period between the trials to avoid the effects of fatigue. The experimental task was performed three times: the initial test (Pre-training 1), five weeks later (Pre-training 2), and one week after completing training (Post-training). The training program began two weeks after the second experimental task and lasted for five weeks.

### Data Collection

All experiments took place at the National Rehabilitation Center for Persons with Disabilities in Saitama, Japan. A seven camera VICON 370 motion analysis system (Oxford Metrics Ink., Oxford, UK) was used to record the 3-D movements of the lower limb. The laboratory was equipped with 2 force plates (9287A; Kistler Japan Co., Ltd., Tokyo, Japan). The motion and force data were recorded at 200 Hz and 1000 Hz, respectively.

For each subject, 24 markers of 9 mm diameter were secured to the lower limb using double-sided adhesive tape as described in a previous study [22]. The markers were used to implement the Point Cluster Technique (PCT) [23]. The PCT provides a minimally invasive estimation of the *in vivo *motion of the knee. By using a cluster system of skin markers on a limb segment, the PCT assumes to cancel out the noise resulted from skin deformation. We developed our algorithm of the PCT following the procedure described by Andriacchi et al. [23]. The knee kinematics, including knee flexion/extension, valgus/varus, and internal/external tibial rotation as well as the anterior/posterior tibial translation, were calculated using the joint coordinate system proposed by Grood and Suntay [24].

Simultaneous electromyographic activity of the rectus femoris (RF), biceps femoris (BF) and semimembranosus (SM) were measured. Pre-amplified surface Ag/AgCl electrodes sensors (DelSys, Inc., Boston, USA) were used to detect muscular activity. A single-ended amplifier (gain = 1000) was used, while the common mode rejection rate was -92 dB. Double-sided adhesive strips were used to adhere the electrodes to the subject's skin. Additionally surgical tape (NICHIBAN Co., Ltd. Tokyo, Japan) was placed over the electrodes as well as around the thigh and shank to retard movement of the electrodes on the skin that would cause movement artifacts. The surface electromyography (EMG) electrodes were placed at the midpoint between the top of the patella and the anterior superior iliac spine over the muscle belly of the RF as well as at a point located distally at one-third of the distance between the knee-joint space and the ischial tuberosity over the muscle bellies of the BF and the SM. A reference electrode was placed on the head of the fibula. The EMG data was recorded at 1000 Hz.

EMG data were recorded 1) while the subjects performed a maximum voluntary knee flexion and extension at 60 degrees of knee flexion against a resistance for three seconds, 2) during a static trial, i.e., while the subjects remained in a standing position for 1 second, and 3) while the subjects performed the single limb drop landing as previously described.

### Training Program

Training lasted approximately 20 minutes a day, 3 days a week for 5 weeks. The program was developed based on a thorough review of the literature [6,25-27] (Table 1). Because the exisiting programs last for considerably long durations, this program was newly designed in order to decrease the training time to 20 minutes. During training, subjects practiced their fundamental basketball skills and additionally carried out jump and balance training to increase their landing skills. During the first three weeks of training, the technique phase (Phase1) which focused on improving the subject's landing technique was implemented. Three basic techniques were stressed: 1) a soft landing with a great bend to the hip, knee and ankle joint; 2) landing on the ball of the foot with the trunk leaning forward; 3) keeping the subject's knee neutral without medial motion. The last two weeks of training, the performance phase (Phase2), focused on increasing the intensity of training and on the use of proper techniques throughout several movements. A trainer or therapist attended the training every time and instructed the athletes to learn these landing techniques throughout each training session.

**Table 1:** Description of the exercises performed during the jump and balance training

Exercise	Time or Repetitions	Exercise	Time or Repetitions
***Phase1: Technique***		***Phase2: Performance***	
1. Squat jumps	20 sec	1. Squat jumps	20 sec
2. 180°jumps	20 sec	2. Scissors jumps	20 sec
3. Single leg balance	20 sec	3. Single leg balance and pass	20 sec
4. Hop jump (both leg)	20 sec	4. Hop jump (single leg)	20 sec
5. Broad jump and hold	28 m	5. Single-leg hop and hold	14 m/leg
6. Crossover hop, hop, hop, stick	28 m	6. Crossover hop, hop, hop, stick	28 m

**Squat jumps**: Drop into deep knee, hip, and ankle flexion and then take off into a maximal vertical jump. On landing, immediately return to the starting position and repeat the initial jump.			

**180°jumps**: Initiates a 2-footed jump with a direct vertical motion combined with a 180°rotation in midair, keeping arms away from the body to help maintain balance. When landing, immediately reverses this jump to the opposite direction.			

**Single-leg balance (and pass)**: This drill is performed on a balance device that provides an unstable surface. Begin by standing on one foot on the device. After the subject has improved, the training drills can incorporate ball catches and passes.			

**Hop jumps**: Start by standing next to a small square balance board. Hop onto the board and then hop off on the opposite side. Repeat hopping on and off the board.			

**Broad jump and hold**: Begin by swinging arms forward and jumping horizontally and vertically at approximately a 45°angle to achieve a maximum horizontal distance. The athlete lands with her knees flexed to approximately 90°.			

**Crossover hop, hop, hop, stick**: Start on a single limb and jump at a diagonal across the body landing on the opposite limb with the foot pointing straight ahead and immediately redirect the jump in the opposite diagonal direction.			

**Scissors jumps**: Start in a stride position with one foot well in front of other. Jump up, alternating foot positions in midair.			

**Single-leg hop and hold**: Initiate the jump by swinging the arms forward while simultaneously extending at the hips and knees. The jump should carry the athlete up at an angle of approximately 45°and attain maximal distance for a single-leg landing. The subject is instructed to land on the jumping leg in deep knee flexion.			

### Data Analysis

The coordinate data obtained from the markers were not smoothed because of the expected noise-canceling property of the PCT. In each trial, we calculated the angular displacements of flexion/extension, valgus/varus, and internal/external tibial rotation using the PCT. The reference position for these measurements was obtained during the static trial. Cerulli et al. [28] reported that ACL strain begins to increase prior to landing and reaches a peak that corresponds to the peak ground reaction force. Therefore, we extracted each variable at the time of foot contact, as well as displacement from the event of foot contact to the event of peak vertical ground reaction force (i.e. during the landing). Moreover, we normalized the event of foot contact to the event of peak vertical ground reaction force as 100%.

The root mean square (RMS) of the EMG data was calculated for each trial. The EMG activities of the two hamstrings muscles were averaged together to represent the whole activity of the hamstrings (Ham). For the maximum voluntary contraction, the EMG data were recorded when the subject performed a maximum voluntary knee flexion and extension at 60 degrees of knee flexion against a manual resistance for three seconds. If the strength of their knee extension was greater than the manual resistance, some research assistants cooperated to provide additional resistance to their motion. The RMS of the EMG data was calculated and the mean RMS of the middle one second was used to normalize the dynamic contraction recorded during the landing (%MVC). The hamstrings/quadriceps (quadriceps refers to the rectus femoris in this experiment) ratio (HQR) was calculated to define the flexor muscle activation relative to the extensor muscle activation. The HQR was calculated according to procedures outline by Bencke et al. [15]. Average %MVC (RF and Ham) and HQR data output was computed during each of the following time frames: 1) 50 ms before foot contact and 2) 50 ms immediately after foot contact. The time period 50 ms before foot contact indicates pre-activity of these muscles. Considering an electromechanical delay of about 50 ms [29], the pre-activation force corresponds to the time before foot contact. The time period 50 ms after foot contact was chosen since a previous study found that the activity of the knee extensors peaked 46 ms after toe contact while the knee flexors showed minimum EMG activity [15]. It is thought that these activities include spinal reflexive neuromuscular activities [30].

The mean kinematics and electromyographic data of each subject were calculated from 3 successful trials. A repeated ANOVA with post-hoc Bonferroni tests was employed for statistical analysis of the kinematics data. Because the %MVC and HQR data did not distribute normally, a Friedman test with Wilcoxon matched-pairs signed rank test was employed for statistical analysis of these data. Each analysis was used to calculate differences with respect to the three time periods. Differences between preliminary trials 1 and 2 were considered as the changes due to the control session; these changes indicate the effects of learning from the landing task and day-to-day individual variation. Differences between preliminary trial 2 and the post-trial were considered as effects of the training session, which indicates how the training program influenced the subjects' landing patterns. All statistical comparisons were performed with the level of significance set at p < 0.05. Intra-class correlation coefficients (ICC (1, 3)), based on multiple same-day trials for kinematics and electromyographic data, and standard error of measurement (SEM) were calculated and are described in Table 2. These data indicate substantial or almost perfect reliability [31].

**Table 2:** ICC (1, 3) and SEM for kinematics and electromyographic data

	Position at foot contact (deg)			
	Flexion	Ext. tibial rot.	Varus	

ICC	0.93	0.94	0.95	
SEM (deg)	1.80	1.42	0.61	

	Displacement during landing (deg)			

	Flexion	Int. tibial rot.	Varus	Valgus

ICC	0.96	0.87	0.64	0.86
SEM (deg)	1.41	2.18	1.22	0.39

	Electromyographic data (%MVC)			

	Rectus femoris		Hamstrings	

ICC	0.79		0.62	
SEM (%)	10.84		9.49	

ICC: Intraclass correlation coefficients

SEM: Standard error of measurement

## Results

### Kinematics Data

**Figure 1:**
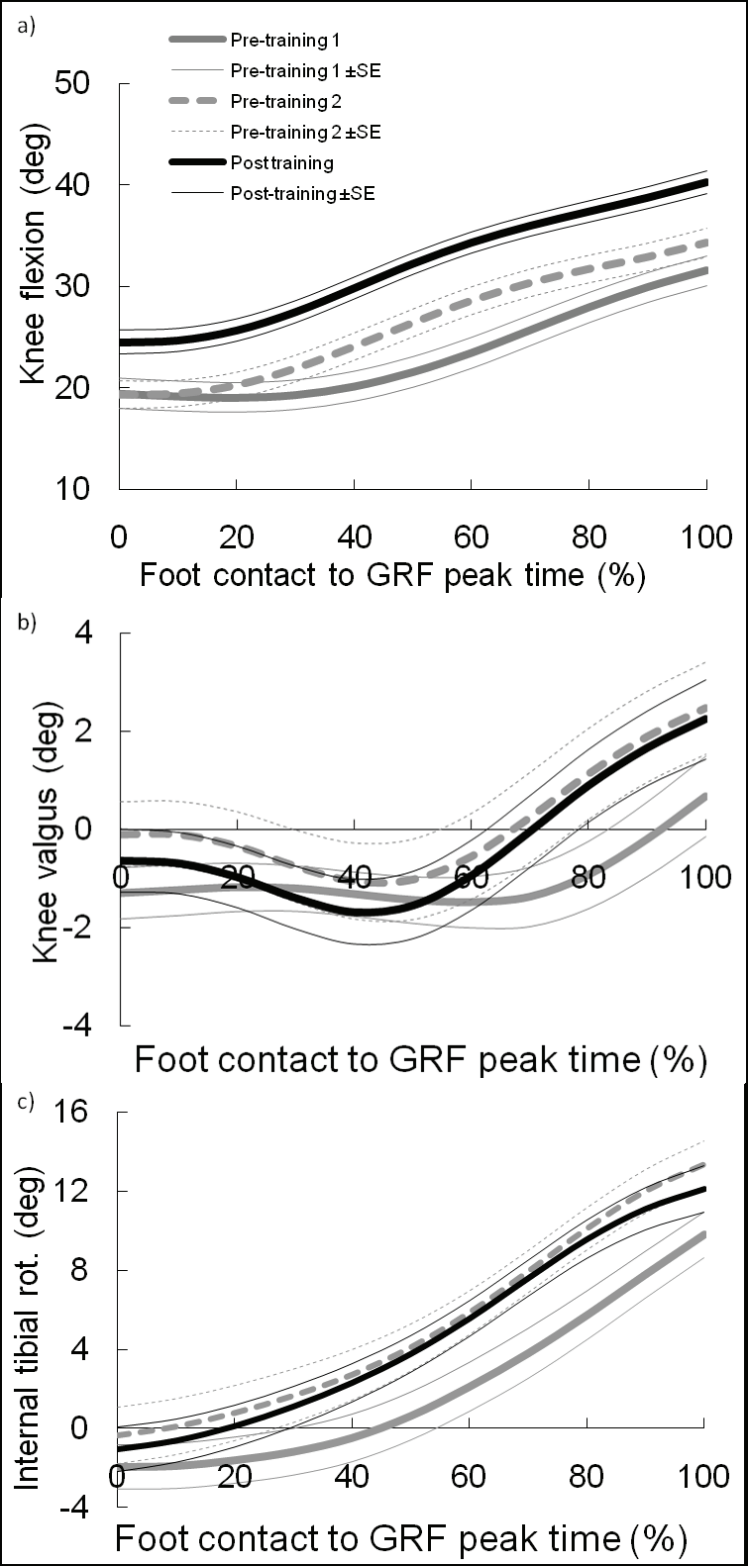
**Mean joint motion across all subjects during single limb drop landing from foot contact to 60 ms after foot contact for Pre-training 1, Pre-training 2, and Post-training**. Data are presented for knee flexion (a), knee valgus (b), and internal tibial rotation (c).

Mean joint kinematics during the single limb drop landing is illustrated in Figure 1. Regarding the position of the knee at foot contact, the knee flexion angle for the Post-training trial was significantly larger than that for the Pre-training 2 trial (p < 0.01) (Table 3). Regarding rotational displacements that occurred during landing, the absolute change in knee flexion for the Post-training trial was significantly larger than that for the Pre-training 2 trial (p < 0.001) (Table 4). No other significant differences between kinematic data could be detected. The observed statistical power of ANOVA for knee flexion, internal tibial rotation, and knee valgus of the tibia at foot contact was 0.96, 0.13, and 0.12, respectively. While, the observed statistical power of ANOVA for the absolute change in knee flexion, internal tibial rotation, and knee valgus during the landing were 1.00, 0.21, and 0.56, respectively.

**Table 3:** Mean (SE) values of position at foot contact Pre-training 1, Pre-training 2, and Post-training (deg)

	Flexion	External tibial rot.	Varus
Pre-training 1	19.5 (2.4)	1.9 (2.2)	1.3 (0.9)
Pre-training 2	19.3 (2.5)*	0.3 (2.5)	0.1 (1.2)
Post-training	24.4 (2.1)*	1.1 (2.0)	0.6 (1.1)

*: p < 0.01 between Pre-training 2 and Post-training

**Table 4:** Mean (SE) values of rotational displacement during the landing Pre-training 1, Pre-training 2, and Post-training (deg)

	Flexion	Internal tibial rot.	Varus	Valgus
Pre-training 1	31.6 (2.5)	12.3 (1.4)	1.4 (0.3)	2.8 (0.6)
Pre-training 2	34.3 (2.5)**	13.8 (1.8)	1.3 (0.3)	3.7 (0.7)
Post-training	40.2 (1.9)**	13.3 (2.0)	1.3 (0.4)	4.1 (0.6)

**: p < 0.001 between Pre-training 2 and Post-training

### Electromyographic Data

**Figure 2:**
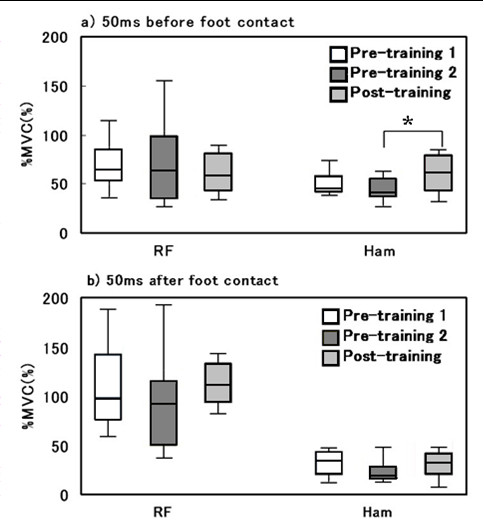
**%MVC of the rectus femoris (RF) and the hamstrings (Ham) for the 50 ms before foot contact (a) and for the 50 ms after foot contact (b)**. Boxes denote the middle 50% of the range and the median. The whiskers show the extent of the rest of the data. * p < 0.05. Figure 2 was quoted by 'The Journal of Clinical Sports Medicine (in Japanese)' 2007, Vol.24: pp 499-503.

**Figure 3:**
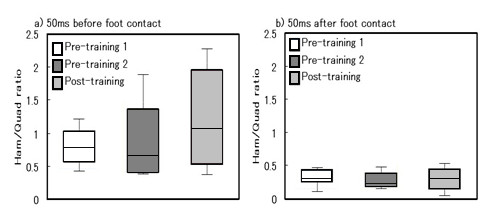
**Ham/Quad-ratio (HQR) before foot contact (a) and after foot contact (b)**. Boxes denote the middle 50% of the range and the median. The whiskers show the extent of the rest of data.

For the 50 ms before foot contact, the %MVC of the Ham in the Post-training trial was significantly greater than that in the Pre-training 2 trial (p < 0.05), while no significant differences could be determined for the RF (Figure 2a). On the other hand, for the 50 ms immediately after foot contact, the %MVC of the Ham and the RF were not significantly different between all trials (Figure 2b). The HQR were not significantly different between all trials for neither the 50 ms before foot contact nor for the 50 ms immediately after foot contact (Figure 3).

## Discussion

This paper examined the effects of a jump and balance training program designed to prevent ACL injury on both knee kinematics and muscle activity during a single limb drop landing. Our training program consisted of plyometrics, jump and balance training, fundamental skills used in basketball and instructions on proper landing techniques. Each training session lasted for only approximately 20 minutes a day, therefore a trainer, coach or therapist could realistically perform this training program easily during practice or warm-up. This jump and balance training program resulted in increased knee flexion and increased hamstrings activity. While we discuss later about each factors; further, multi-component training, including jump and balance training could result in changes in knee kinematics and femoral muscle activity.

The training program was considered to influence knee flexion during landing. However, in tibial rotation and knee varus/valgus, the difference between the training sessions was smaller than the SE and learning effects (difference from the control session). Therefore, the changes in tibial rotation and knee varus/valgus were not considered to be effects of the training program. While the results regarding knee flexion partially proved our hypothesis, our hypotheses that training would decrease knee valgus and internal tibial rotation were not supported. For frontal plane knee motion previous studies [11,14] reported no differences in knee valgus during single limb landing after plyometric or agility training. However, Myer et al. [12] reported that both plyometric and balance training decreased the knee abduction angle during a medial drop landing. There is a possibility that single limb drop landing is not suitable to detect the change in frontal plane knee motion. It is also thought that the number of subjects in this study was low and the statistical power for the valgus. This low statistical power makes it difficult to demonstrate the small changes in knee valgus. For tibial rotation, although we could not compare with previous studies, it is possible that these same reasons impede detecting the change in motion in knee frontal motion (i.e. selection of task and statistical problem). Otherwise, it may be difficult to control tibial rotation, like joint laxity.

According to other studies that used video analysis, the position of the knee at the moment of injury is in slight knee flexion and valgus rotation with either internal or external tibial rotation [19-21]. Teitz [19] reported that the angle of knee flexion at the time of injury was less than 30 degrees. In this study, slight knee flexion, valgus and internal tibial rotation also occurred during the single limb drop landing. It is thought that decreasing tibial rotation and valgus rotation or increasing the angle of knee flexion is effective in preventing this knee position at the moment of ACL injury. Some previous studies [11,12] indicated that training increases the knee flexion angle during both limb landings. The results of this study agree with those previous studies. Onate et al.[13] reported that videotape feedback and instruction increased peak knee flexion during landing. In this study, subjects increased not only peak knee flexion, but also initial knee flexion at foot contact. Because subjects were instructed to go through more knee flexion during training, increased peak knee flexion was not a novel finding. However, continuing the jump and balance training over a period of time also increased initial knee flexion. Thus it can be said that these increases in knee flexion are a direct effect of the jump and balance training.

Increased knee flexion angles may lead to changes in the function of the quadriceps and the hamstrings. In slight knee flexion, i.e., less than 30 degrees, contraction of the quadriceps strains the ACL [32-34]. Hamstrings contraction cannot reduce ACL strain with the knee slightly flexed because these muscles meet the tibia at a small angle [33,35]. On the other hand, at angles of knee flexion greater than 60 degrees, quadriceps contraction does not increase ACL strain [34] and the anterior tibial translation and internal tibial rotation as a result of quadriceps contraction is decreased [36]. Moreover, hamstrings contraction reduces anterior tibial translation and internal tibial rotation at these angles [37]. Therefore, increasing the knee flexion angle during landing may have a beneficial effect to reduce the strain in the ACL. From the results of this study, the jump and balance training program which increased the knee flexion angle during landing also had the effect of decreasing the risk of ACL injury.

Hamstrings activity was also increased after training. In the training sessions, subjects were instructed to bend their knees and keep their knees neutral. Subjects were directed to keep a deep knee flexion angle during landing and to stabilize the position of their knee joint on the balance board. It is reported that peak activity of the hamstrings occur between 50-70 degrees of knee flexion [38]. Hamstrings contraction and coactivation of the hamstrings and the quadriceps have an important role to stabilize the knee joint [37,39-41]. Therefore, the subjects learned the correct posture and landing technique which would increase hamstrings activity.

The activity of the hamstrings before foot contact was significantly increased. Recently several researchers have focused on pre-activated muscle patterns in response to anticipated movements and joint loads [15,42]. Pre-activation is important for the dynamic stability of the knee because it provides fast compensation for encountered external loads. Wojtys and Huston [30] described the possibility that pre-activation can provide adequate muscular protection to restrain the knee. Although there are several reflexes that occur after a perturbation [40], the muscle activity is too slow to provide any ligament protection [43,44]. Between preparatory and reactive muscle activation, there is a period of latency that results from electromechanical delay (EMD) [29]. EMD is the delay between neural stimulation of a muscle and the development of muscle tension. Pre-activation is thought to be beneficial for stabilizing the knee after a landing. For this study, although internal tibial rotation and tibial translation were not changed after training, it can be said that the increase in the pre-activity of the hamstrings helped to stabilize the knee joint during landing.

There are some limitations in this study. First, we analyzed only the kinematics of the knee joint, even though hip and ankle joint kinematics also play an important role during landing. We examined only the short term effects of the ACL injury prevention training. The incidence of ACL injury during the season was not investigated. We examined the effects of multi-component training. The effects of each training should be examined in the future. We did not consider the effects of menstrual cycle. Our study included control and training sessions, although setting up a control group and an intervention group is ideal to examine the effects of training. However, there were no changes in basketball training during these sessions, which were conducted during the preseason, and training and matches occurred at the same frequency during both sessions. Therefore, the changes in kinematics during the landing could be attributable to the intervened training. Finally, the statistical power for several of the data was relatively low. Using ANOVA at a low statistical power might cause type 2 errors. Although considerable differences in results might be significant, other findings may be overlooked. Future research should be performed to examine the long term effects of the ACL injury prevention program in a large sample size.

## Conclusions

The results of this study indicate that the jump and balance training increased knee flexion and hamstrings activity in female basketball athletes during a single limb drop landing. This program, which includes plyometrics, jump and balance training, fundamental basketball skills, and proper landing instructions, might have partial effects that avoid the characteristic knee position of ACL injury, thereby preventing injury. However, the expected changes in frontal and transverse kinematics of the knee were not observed.

## Competing interests

Grant-in-Aid for Scientific Research (C) (16500394)

## Authors' contributions

YN participated in the design of the study and drafted the manuscript. HI developed an algorithm of the PCT. MA and TF conceived of the study, and participated in its design and coordination and helped to draft the manuscript. Each author has read and concurs with the final manuscript's contents.
